# Supplementary material for: Combined BRCA2 and MAGEC3 Expression Predict Outcome in Advanced Ovarian Cancers
Source: Cancers (Basel). 2022 Sep 28;14(19):4724. doi: 10.3390/cancers14194724 (PMC9562635; doi:10.3390/cancers14194724)
Supplement: Supplementary file 1 [file cancers-14-04724-s001.zip › cancers-1898083-supplementary.pdf]

Combined BRCA2 and MAGEC3 expression predict outcome in Advanced Ovarian Cancers

-

Supplementary Figures

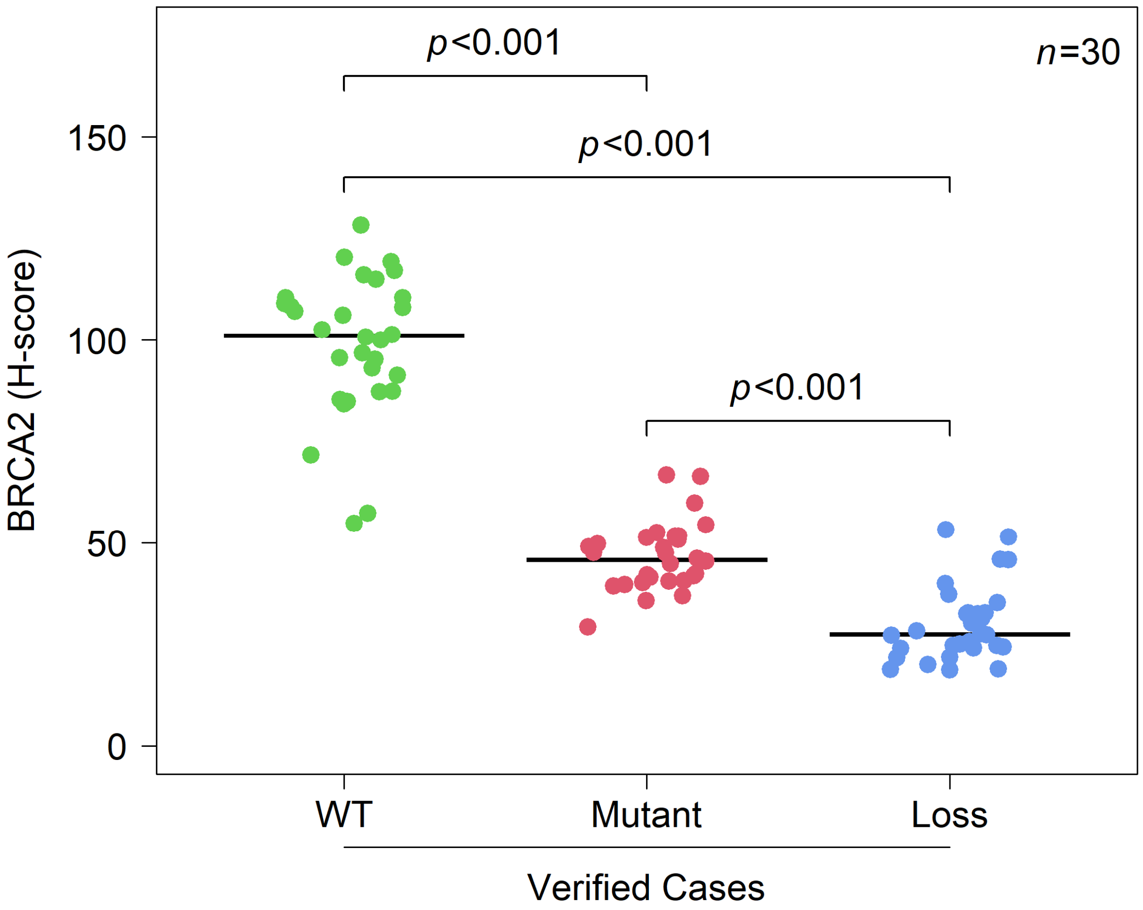

Figure S1. BRCA2 N-terminal expression in the sequenced ovarian tumor cases. A statistically significant difference observed between in the N-terminal expression of BRCA2 wild type, BRCA2 mutant, and BRCA2 loss in sequenced ovarian tumor cases when compared to each other.

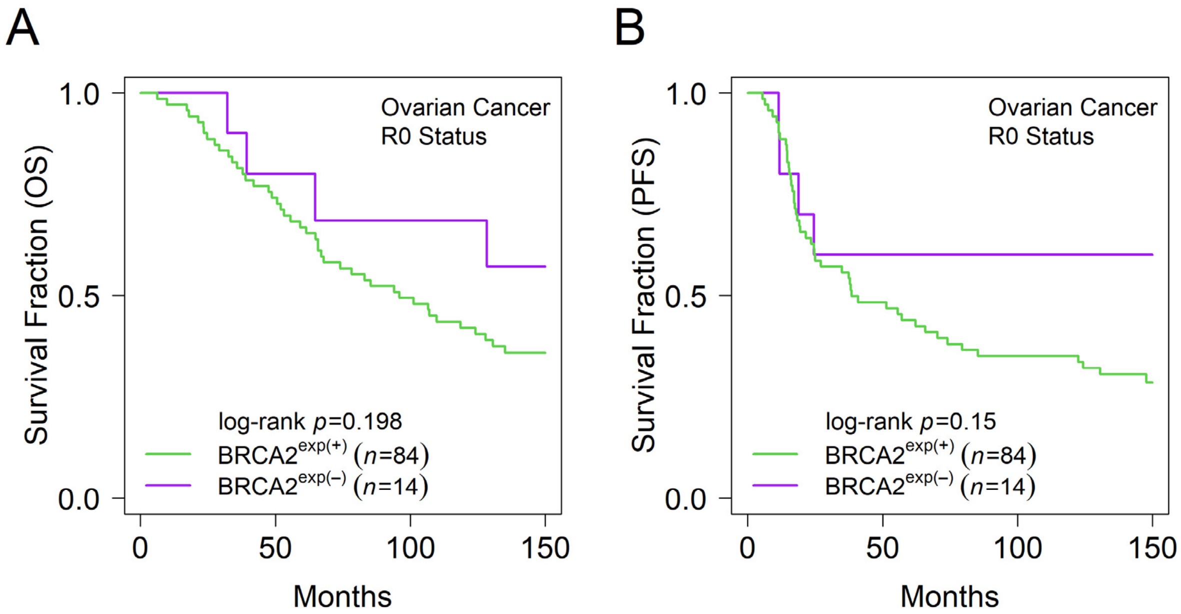

Figure S2. Kaplan–Meier survival curves for independent evaluation of the relationship between BRCA2 expression and patient outcomes in the larger cohort of patients with unsequenced advanced ovarian cancer cases. (**A**) Kaplan–Meier plot showing no improvement in overall survival trends for advanced ovarian cancer patients stratified by BRCA2 expression levels. (**B**) Kaplan–Meier plot showing no improvement in progression-free survival trends for advanced ovarian cancer patients stratified by BRCA2 expression levels.
